# A Sign of the Times

**Published:** 1911-08

**Authors:** 


A SIGN OF THE TIMES.—"As a sign of the times, it is herewith recorded that a bill providing that men applying for a marriage license shall undergo a physical examination was passed in the House of the Indiana Legislature. Other State assemblies are wrestling with the question of enacting such a law, and everywhere it is evident that the general public is increasing in intelligence. With a broadening of the mind comes an increased moral recognition of the essentials of good government.—*Lancet-Clinic.*

This is along the line of progress. If society has the right to protect itself from the depredations of the criminal, why has it not the right to use prophylaxis in the same direction? We deny to other nations the privilege of dumping their paupers and criminal class upon our shores,—why may we not with equal right deny to the physically unfit the privilege of propagating their kind? It is unquestionably true that the criminal class is recruited from the offspring of physically diseased parents. We are coming to believe more and more that crime is but a manifestation of disease, chiefly of the nervous system. Given a healthy parentage and the coming generation would cut crime to a minimum. If the diseased could be denied the right to produce children, we could turn our jails into factories and our alms houses into seminaries.

But why stop with refusing to grant a marriage license? It is fairly well established that marriage is not absolutely necessary to the begetting of children. If a man or a woman is not fit to receive a marriage license, and the condition is not amenable to treatment, he or she should be sterilized. It is only by such a procedure that society can be sure that these unfortunates are not going to do their best to populate the earth with similar beings.—*Editorial, Medical Sentinel.*

